# First-in-human phase I/II, open-label study of the anti-OX40 agonist INCAGN01949 in patients with advanced solid tumors

**DOI:** 10.1136/jitc-2021-004235

**Published:** 2022-10-31

**Authors:** Elizabeth J Davis, Juan Martin-Liberal, Rebecca Kristeleit, Daniel C Cho, Sarah P Blagden, Dominik Berthold, Dana B Cardin, Maria Vieito, Rowan E Miller, Prashanth Hari Dass, Angela Orcurto, Kristen Spencer, John E Janik, Jason Clark, Thomas Condamine, Jennifer Pulini, Xuejun Chen, Janice M Mehnert

**Affiliations:** 1 Vanderbilt University Medical Center, Nashville, Tennessee, USA; 2 Vall d'Hebron Institute of Oncology (VHIO), Barcelona, Spain; 3 Research Department of Oncology, University College London, London, UK; 4 Perlmutter Cancer Center, NYU Langone Health, NYU Grossman School of Medicine, New York, New York, USA; 5 Department of Oncology, University of Oxford, Oxford, UK; 6 Department of Oncology, Centre Hospitalier Universitaire Vaudois (CHUV), Lausanne, Switzerland; 7 University College London Hospital, London, UK; 8 Early Phase Clinical Trials Unit, Churchill Hospital, University of Oxford, Oxford, UK; 9 Rutgers University, New Brunswick, New Jersey, USA; 10 Incyte Corporation, Wilmington, Delaware, USA

**Keywords:** Clinical Trials as Topic, Immunomodulation, Lymphocytes, Tumor-Infiltrating, T-Lymphocytes, Tumor Microenvironment

## Abstract

**Background:**

OX40 is a costimulatory receptor upregulated on antigen-activated T cells and constitutively expressed on regulatory T cells (Tregs). INCAGN01949, a fully human immunoglobulin G1κ anti-OX40 agonist monoclonal antibody, was designed to promote tumor-specific immunity by effector T-cell activation and Fcγ receptor-mediated Treg depletion. This first-in-human study was conducted to determine the safety, tolerability, and preliminary efficacy of INCAGN01949.

**Methods:**

Phase I/II, open-label, non-randomized, dose-escalation and dose-expansion study conducted in patients with advanced or metastatic solid tumors. Patients received INCAGN01949 monotherapy (7–1400 mg) in 14-day cycles while deriving benefit. Safety measures, clinical activity, pharmacokinetics, and pharmacodynamic effects were assessed and summarized with descriptive statistics.

**Results:**

Eighty-seven patients were enrolled; most common tumor types were colorectal (17.2%), ovarian (8.0%), and non-small cell lung (6.9%) cancers. Patients received a median three (range 1–9) prior therapies, including immunotherapy in 24 patients (27.6%). Maximum tolerated dose was not reached; one patient (1.1%) receiving 350 mg dose reported dose-limiting toxicity of grade 3 colitis. Treatment-related adverse events were reported in 45 patients (51.7%), with fatigue (16 (18.4%)), rash (6 (6.9%)), and diarrhea (6 (6.9%)) being most frequent. One patient (1.1%) with metastatic gallbladder cancer achieved a partial response (duration of 6.3 months), and 23 patients (26.4%) achieved stable disease (lasting >6 months in one patient). OX40 receptor occupancy was maintained over 90% among all patients receiving doses of ≥200 mg, while no treatment-emergent antidrug antibodies were detected across all dose levels. Pharmacodynamic results demonstrated that treatment with INCAGN01949 did not enhance proliferation or activation of T cells in peripheral blood or reduce circulating Tregs, and analyses of tumor biopsies did not demonstrate any consistent increase in effector T-cell infiltration or function, or decrease in infiltrating Tregs.

**Conclusion:**

No safety concerns were observed with INCAGN01949 monotherapy in patients with metastatic or advanced solid tumors. However, tumor responses and pharmacodynamic effects on T cells in peripheral blood and post-therapy tumor biopsies were limited. Studies evaluating INCAGN01949 in combination with other therapies are needed to further evaluate the potential of OX40 agonism as a therapeutic approach in patients with advanced solid tumors.

**Trial registration number:**

NCT02923349.

**WHAT IS ALREADY KNOWN ON THIS TOPIC**

- OX40 agonist antibodies have been shown to reduce tumor growth in preclinical models, but the benefit of these agents in clinical studies has not been demonstrated to date.

**WHAT THIS STUDY ADDS**

- This phase I/II, first-in-human study demonstrates that monotherapy with the OX40 agonist antibody INCAGN01949 was generally well tolerated, with one patient achieving a partial response and 23 achieving stable disease (SD) among 87 enrolled patients with advanced solid tumors. This study adds to published literature reporting patients achieving SD with other OX40 agonist antibodies; however, objective responses are rarely observed in the advanced cancer setting.

**HOW THIS STUDY MIGHT AFFECT RESEARCH, PRACTICE OR POLICY**

- Optimizing the therapeutic potential of OX40 agonists may require novel combinations and/or sequential dosing strategies, or evaluation in patients with immunologically responsive tumors.

## Introduction

Immune cell receptors modulate key cellular functions that typically require activation or inhibition of signaling pathways to regulate immune responses.^1–4^ Immune checkpoint receptors have been shown to negatively regulate antitumor immune responses in the tumor microenvironment, such as cytotoxic T-lymphocyte antigen 4 (CTLA-4, expressed on activated cytotoxic T cells and regulatory T cells (Tregs)), programmed death-ligand 1 (PD-L1; expressed on activated dendritic cells, macrophages, and tumors), and programmed cell death protein 1 (PD-1; expressed on activated T and B cells, natural killer cells, and antigen-presenting cells).^5^ The identification of inhibitory and stimulatory immune receptor targets has led to the development of immune receptor-specific monoclonal antibodies as potential targeted immunotherapies for the treatment of cancer.^4 6 7^

Antitumoral immunity may also be achieved by agonist antibody activation of T-cell costimulatory receptors, which mostly belong to the tumor necrosis factor receptor superfamily (TNFRSF).^5 8^ OX40 (also known as CD134 or TNFRSF4) has been shown to positively regulate lymphocyte activation, proliferation, and apoptosis, depending on the tumor microenvironment.^9 10^ Expression of OX40 has been detected on activated T cells, including CD4^+^ and CD8^+^, and is constitutively expressed on Tregs.^11^ OX40^+^ T cells have been found in tumor samples from patients with melanoma, cutaneous squamous cell carcinoma, head and neck squamous cell carcinoma, and breast, colon, gastric, and ovarian cancers.^12–18^ Within inflammatory lesions, OX40 has been shown to be transiently expressed, being upregulated on the most recently antigen-activated T cells (eg, tumor-infiltrating lymphocytes (TILs)).^9^ In preclinical models, OX40 agonist antibody reduced tumor growth, with an overall increase in the number and activity of effector T cells (CD4^+^ and CD8^+^), and concomitant decrease in Tregs (forkhead box protein 3 (FoxP3)^+^).^19 20^

INCAGN01949 is a fully human immunoglobulin G1κ anti-OX40 agonist monoclonal antibody being investigated for the treatment of advanced malignancies and has two potential mechanisms of action. First, targeting OX40 with INCAGN01949 and activating NFκB-mediated costimulation signaling could enhance expansion, differentiation, and survival of tumor-specific effector and memory T cells.^8 21^ OX40 signaling may also prevent Treg-mediated suppression of antitumor immune responses.^8^ Second, INCAGN01949 has intact Fc receptor function that may promote antibody-dependent cellular cytotoxicity to selectively deplete intratumoral Tregs and limit their immunosuppressive effects in the tumor microenvironment, similar to what has been reported for antibodies against CTLA-4.^22 23^ Based on the potential mechanisms of action, treatment with INCAGN01949 is anticipated to enhance intratumoral immune activation, with an increase in effector T cell to Treg ratio. To assess effects of INCAGN01949, pharmacodynamic analyses such as investigating increases in peripheral blood T-cell activity could potentially become correlative measures of immune cell activation.^13^

Here we report results of a first-in-human, phase I/II study conducted to determine the safety and tolerability of INCAGN01949, define the maximum tolerated dose (MTD) or pharmacologically active dose (PAD), and assess preliminary efficacy in patients with advanced solid tumors. Pharmacodynamic analyses were also conducted to assess potential antitumor activity of INCAGN01949.

## Methods

### Study design

This phase I/II, open-label, non-randomized, dose-escalation and dose-expansion study was conducted between November 29, 2016, and March 26, 2019.

**Figure 1:**
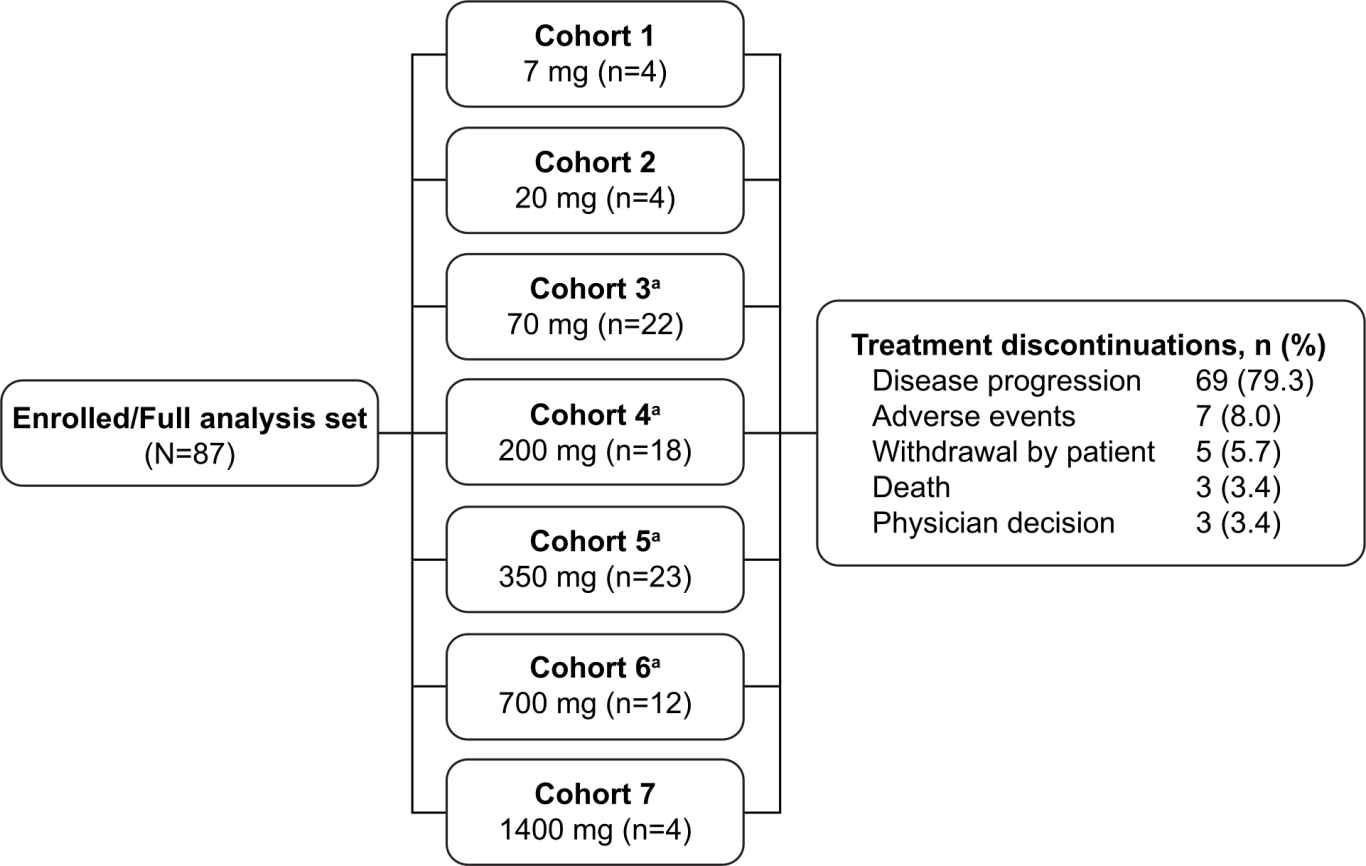
Patient disposition. ^a^Cohorts included patients enrolled in dose-escalation and safety-expansion populations. All doses (7–1400 mg) refer to INCAGN01949.

The study was designed to be conducted in two parts, a 3+3 treatment dose escalation with a safety expansion (part 1) and a dose expansion (part 2) to explore the preliminary safety and efficacy in specific tumor types. Patients received INCAGN01949 as a 30 min intravenous infusion on day 1 of each 14-day cycle (14±3 days). Dosing was fixed as shown in figure 1. In part 1, patients received INCAGN01949 and dose escalation started at a dose of 7 mg. There was a 48-hour safety waiting period between dosing of the first and second patients of each cohort, and the first three patients of a cohort were observed for dose-limiting toxicities (DLTs, online supplemental table S1) for 28 days before the next cohort began treatment. DLTs included non-hematological (grade ≥3), hematological (grade ≥3), immune-related (grade ≥2 (ocular) or grade ≥3), and general (inability to receive planned number of doses within 28-day DLT period, regardless of grade). If patients dropped out of the study for reasons other than a DLT, they were replaced to have a minimum of three evaluable patients per cohort. Intercohort dose escalation then proceeded independently until a PAD or MTD was determined. The PAD was defined as a dose that provides a maximal biochemical effect or an increase in biomarkers of immune activity; the MTD was defined as one dose level below a dose at which greater than one-third of patients in a cohort have a DLT.

10.1136/jitc-2021-004235.supp1 Supplementary data

A safety expansion (maximum 36 patients) was included in part 1 to confirm the preliminary safety findings observed in the dose-escalation cohorts, further evaluate the pharmacodynamic activity of INCAGN01949, and determine a recommended dose/schedule. A cohort was considered safe if less than three of nine evaluable patients reported a DLT. If more than one safety-expansion cohort was considered safe, the recommended dose and schedule were determined by the investigators and sponsor, based on available safety, pharmacokinetic, pharmacodynamic, and biomarker findings. Patients could continue to receive INCAGN01949 in 14-day cycles while deriving benefit (no clinical progression as defined by two confirmatory scans) and not meeting protocol-defined conditions for treatment withdrawal (including withdrawn consent, investigator judgment, unacceptable toxicity, pregnancy, lost to follow-up, and study termination).

### Study population

Eligible patients must have been ≥18 years of age with advanced or metastatic solid tumors, disease progression while on or intolerant to standard therapies, an Eastern Cooperative Oncology Group performance status ≤1, measurable disease by Response Evaluation Criteria in Solid Tumors (RECIST) v1.1, and provided consent for pretreatment and on-treatment tumor biopsies. An immune-responsive tumor type was not an inclusion requirement. Patients were excluded if they had received previous OX40 agonist treatment for any indication, prior treatment with chemotherapy, targeted small-molecule therapy or radiotherapy within 14 days, monoclonal antibody (except denosumab), immunotherapy or persistence of active cellular therapy within 28 days, immunosuppressive therapy within 7 days, or live vaccine within 30 days, had persistent grade 2 or above toxicity from prior therapy and/or surgical complication, or had active autoimmune disease, central nervous system metastases, and/or carcinomatous meningitis.

### Study endpoints

Safety and tolerability of INCAGN01949 were the primary endpoints, assessed by monitoring the frequency, duration, and severity of adverse events (AEs). Severity of AEs was graded based on the National Cancer Institute Common Terminology Criteria for AEs V.4.03 and summarized using Medical Dictionary for Regulatory Activities v19.1 preferred terms. The main secondary endpoint was the pharmacokinetics of INCAGN01949, including the minimum and maximum (C_max_) plasma drug concentrations, time to maximum plasma drug concentration (T_max_), and area under the concentration–time curve up to the last measurable concentration (AUC_0–t_), analyzed after the first dose of study drug and at steady state.

Other secondary endpoints included best objective response rate defined as sum of complete responses (CRs) and partial responses (PRs) obtained as best response, progression-free survival, duration of response, and duration of disease control defined as CR, PR, or stable disease (SD), as assessed by the investigator using RECIST v1.1 and modified RECIST v1.1. Radiological assessments were performed at baseline, every 8 (±1) weeks during treatment, at the end of treatment (±3 days), and safety follow-up visits (30 (+7) days and 60 (+7) days after the end of treatment).

Assessment of immunogenicity (defined as the occurrence of specific antidrug antibody (ADA) measurements taken before treatment on day 1 of cycles 1, 2, 3, and 6, as well as a follow-up safety visit) was an exploratory endpoint.

### Correlative translational studies

Correlative translational analyses performed as exploratory study endpoints included correlation between plasma levels of INCAGN01949 and receptor occupancy (using an OX40-expressing cell model), and effects of INCAGN01949 on biomarkers in peripheral blood and tumor tissue (collected by biopsy at cycles 3–4) and compared with baseline.

#### OX40 receptor occupancy analysis

Jurkat cells overexpressing OX40 receptor were incubated for 30 min with patients’ plasma (baseline and on-treatment). Saturated concentrations of fluorescently labeled INCAGN01949 were added, and cells were incubated for 15 min; cells were then washed and analyzed for fluorescence by flow cytometry.

#### Whole blood T-cell population profiling

Baseline and on-treatment immune cell frequency were monitored by flow cytometry analysis of T-cell subsets (memory/naïve and Treg) and T-cell function (activation/exhaustion) by Caprion Biosciences (Montreal, Canada). T-cell marker panels identified proliferating T cells (Ki67^+^ CD3^+^), Treg cells (CD3^+^ CD4^+^ CD25^+^ CD127^−^ FoxP3^+^), and activated/exhausted T cells (PD-1^+^ CD3^+^).

#### T-cell function analysis

To evaluate T cell function, cryopreserved peripheral blood mononuclear cells were restimulated at Isoplexis (Branford, USA) and cytokine production was analyzed at a single-cell level. Briefly, CD4^+^ and CD8^+^ T cells were isolated using magnetic bead separation techniques and stimulated with anti-CD3 and anti-CD28 antibodies for 16 hours at 37°C. After the incubation time, cells were recovered and loaded onto Isoplexis IsoLight System. Cytokine secretion at a single-cell level was analyzed for 32 cytokines by ELISA-based technology. Polyfunctional T-cell frequency (cells secreting ≥2 cytokines) and polyfunctional strength index (which combines the polyfunctionality of a sample (frequency of cells secreting multiple cytokines) with the signal intensities for each single cell across the secreted cytokines of the sample) were calculated for each sample.

#### Plasma protein marker analysis

Immune and non-immune modulation plasma proteins were determined using a multiplex Proximity Extension Assay (Olink Proteomics, Watertown, USA). Proteins were identified by a matched pair of antibodies coupled to unique, partially complementary oligonucleotides and measured by quantitative real-time PCR. Paired baseline and on-treatment samples (38 patients) were evaluated for >1100 plasma analytes including soluble OX40 (TNFRSF4), monocyte chemoattractant protein-1, AXL, growth arrest-specific 6, CD93, matrix metalloproteinase-2 (MMP-2), and MMP-3.

#### Transcriptomic analysis

Ten paired biopsy samples that were available from patients enrolled in the study were analyzed by RNA sequencing at BGI Genomics (Cambridge, USA). Briefly, tumor region was annotated by a certified pathologist and macrodissected. RNA was isolated from the tumor region and quality of the extracted RNA was assessed. Libraries were prepared from extracted RNA using TruSeq RNA Access Library Prep kit (Illumina, San Diego, USA). Sequencing was performed on a Hiseq 2500 system (Illumina). Raw data sequencing were aligned and normalized using Incyte internal analysis pipeline.

### Statistical analysis

For reporting purposes, the dose-escalation cohorts and safety-expansion cohorts were combined and summarized by dose level. Safety was assessed in the full analysis set, which included all patients enrolled in the study who received at least one dose of INCAGN01949. Safety measures, including treatment-emergent adverse events (TEAEs), clinical laboratory values, vital signs, and electrocardiograms were summarized with descriptive statistics. Pharmacokinetic parameters were summarized by part, dose, and study cycle. Pharmacodynamic data were analyzed using summary statistics, and biomarker effects were summarized descriptively. Effects of treatment on plasma protein biomarkers were determined by paired t-test comparing baseline (C1D1) values to on-treatment values (C1D2, C1D7 and/or C2D1), with a difference deemed significant at a false discovery rate p value of <0.05. SAS v9.4 (SAS Institute Inc, Cary, USA) was used to generate all tables, graphs, and statistical analyses.

## Results

### Baseline characteristics, patient disposition, and exposure to treatment

Eighty-seven patients were enrolled in the study at eight sites in Spain, Switzerland, the UK, and the USA. Baseline patient characteristics are summarized in table 1. Median age was 60 (range 20–79) years; 60.9% of the patients were female, and 90.8% were white. The most common tumor type was colorectal cancer (17.2%), followed by ovarian (8.0%), non-small cell lung cancer (6.9%), and melanoma and pancreatic cancers (each 5.7%). The majority of patients enrolled (63 (72.4%)) had metastatic disease; most (85 (97.7%)) had received prior therapy with a median of 3 (range 1–9) therapies, and 24 patients (27.6%) had received prior immunotherapy (all except for one patient had immunotherapy that included PD-1/PD-L1 inhibition).

**Table 1:** Baseline demographics and characteristics

Characteristics	Treatment group (dose level)							
	7 mg (n=4)	20 mg (n=4)	70 mg (n=22)	200 mg (n=18)	350 mg (n=23)	700 mg (n=12)	1400 mg (n=4)	Total (N=87)
Median age, years (range)	58 (32–69)	67 (47–74)	56 (27–78)	61 (25–79)	60 (35–78)	62 (37–73)	56 (20–79)	60 (20–79)
Sex, n (%)								
Female	2 (50.0)	3 (75.0)	14 (63.6)	12 (66.7)	17 (73.9)	3 (25.0)	2 (50.0)	53 (60.9)
Male	2 (50.0)	1 (25.0)	8 (36.4)	6 (33.3)	6 (26.1)	9 (75.0)	2 (50.0)	34 (39.1)
Race, n (%)								
White	4 (100)	3 (75.0)	19 (86.4)	17 (94.4)	23 (100)	9 (75.0)	4 (100)	79 (90.8)
Other	0	1 (25.0)	3 (13.6)	1 (5.6)	0	3 (25.0)	0	8 (9.2)
Tumor type, n (%)								
Colorectal cancer	3 (75.0)	0 (0.0)	1 (4.5)	3 (16.7)	6 (26.1)	1 (8.3)	1 (25.0)	15 (17.2)
Ovarian	0	0	3 (13.6)	2 (11.1)	1 (4.3)	1 (8.3)	0	7 (8.0)
Non-small cell lung cancer	0	1 (25.0)	2 (9.1)	1 (5.6)	1 (4.3)	1 (8.3)	0	6 (6.9)
Melanoma	0	0	1 (4.5)	2 (11.1)	2 (8.7)	0	0	5 (5.7)
Pancreatic cancer	0	1 (25.0)	0	0	3 (13.0)	1 (8.3)	0	5 (5.7)
Anal cancer	0	0	1 (4.5)	0 (0.0)	0 (0.0)	1 (8.3)	2 (50.0)	4 (4.6)
Head and neck cancer	0	0	1 (4.5)	1 (5.6)	1 (4.3)	1 (8.3)	0 (0.0)	4 (4.6)
Sarcoma	0	0	2 (9.1)	1 (5.6)	0	0	1 (25.0)	4 (4.6)
Other solid tumors*	1 (25.0)	2 (50.0)	11 (50.0)	8 (44.4)	9 (39.1)	6 (50.0)	0	37 (42.5)
Received prior therapy, n (%)	4 (100)	4 (100)	21 (95.5)	18 (100)	23 (100)	11 (91.7)	4 (100)	85 (97.7)
Advanced/metastatic regimen	4 (100)	4 (100)	21 (95.5)	18 (100)	21 (91.3)	11 (91.7)	4 (100)	83 (95.4)
Surgery	4 (100)	2 (50.0)	18 (81.8)	15 (83.3)	17 (73.9)	9 (75.0)	3 (75.0)	68 (78.2)
Radiotherapy	2 (50.0)	1 (25.0)	13 (59.1)	10 (55.6)	10 (43.5)	8 (66.7)	4 (100)	48 (55.2)
Immunotherapy^†^	0	2 (50.0)	6 (27.3)	7 (38.9)	7 (30.4)	2 (16.7)	0	24 (27.6)

*Mesothelioma (three patients, 3.4%); bladder, breast, cervical, endometrial, esophageal, gallbladder, hepatocellular, neuroendocrine, and testicular (two patients, 2.3% each); adrenal, chordoma, gastroesophageal, renal, salivary gland, and uterine (one patient, 1.1% each); other cancers (10 patients, 11.5%).

†Twenty-three patients (26.4%) received immunotherapy that included PD-1/PD-L1 therapy; one patient (1.1%) in the 20 mg cohort received antivascular endothelial growth factor receptor-2 (ramucirumab) immunotherapy (did not receive any PD-1/PD-L1 therapy).

PD-1, programmed cell death protein 1; PD-L1, programmed death-ligand 1.

Patients enrolled received 7 mg (n=4, cohort 1), 20 mg (n=4, cohort 2), 70 mg (n=22, cohort 3), 200 mg (n=18, cohort 4), 350 mg (n=23, cohort 5), 700 mg (n=12, cohort 6), and 1400 mg (n=4, cohort 7) of INCAGN01949 (figure 1). Cohorts 3–6 of the study included patients enrolled in the dose escalation and safety expansion. Following discussion between investigators and the sponsor, doses of INCAGN01949 tested for expansion were 70, 200, 350, and 700 mg.

One patient in the 350 mg dose cohort experienced a DLT of colitis resulting in discontinuation of the treatment. TEAEs led to treatment interruption in 14 patients (16.1%) and discontinuation in 7 patients (8.0%; ataxia, blood bilirubin increase, cancer pain, cauda equina syndrome, colitis, intestinal obstruction, and spinal cord compression (1 patient each)). All patients discontinued study treatment, most commonly owing to disease progression (79.3%) (figure 1). The number of treatment cycles across cohorts were similar with a median of 4.0 (range 1–18) cycles in the overall (full analysis set) population. The median duration of treatment was 43.0 (range 1–273) days.

### Safety

Overall, 83 patients (95.4%) had any-grade TEAEs, including 45 (51.7%) who had treatment-related adverse events (TRAEs). TEAEs occurring in ≥5% of patients are summarized in table 2. TRAEs occurring in ≥5% of patients were fatigue (n=16 [18.4%]), rash (n=6 [6.9%]), and diarrhea (n=6 [6.9%]). There were no clinically significant trends or signals in any hematology or clinical chemistry parameters. One patient experienced worsening of hematology values (lymphocytes increase) to grade ≥4 in severity; three patients (each with lipase and urate increase) and one patient (with amylase increase) experienced worsening of clinical chemistry values to grade ≥4 in severity. No clinically meaningful trends were observed in vital signs.

**Table 2:** Treatment-emergent adverse events occurring in >5% of the total patients (N=87) treated with INCAGN01949 (all grades and grade ≥3)

Preferred term, n (%)	Treatment group (dose level)							Total (N=87)
	7 mg (n=4)	20 mg (n=4)	70 mg (n=22)	200 mg (n=18)	350 mg (n=23)	700 mg (n=12)	1400 mg (n=4)	
Fatigue	2 (50.0)	2 (50.0)	6 (27.3)	7 (38.9)	8 (34.8)	4 (33.3)	2 (50.0)	31 (35.6)
Grade ≥3	0	1 (25.0)	2 (9.1)	0	0	0	0	3 (3.4)
Decreased appetite	1 (25.0)	3 (75.0)	6 (27.3)	4 (22.2)	7 (30.4)	3 (25.0)	0	24 (27.6)
Grade ≥3	0	0	0	0	0	0	–	0
Constipation	1 (25.0)	1 (25.0)	4 (18.2)	6 (33.3)	4 (17.4)	1 (8.3)	1 (25.0)	18 (20.7)
Grade ≥3	0	0	0	0	0	0	0	0
Vomiting	1 (25.0)	0	6 (27.3)	4 (22.2)	5 (21.7)	0	0	16 (18.4)
Grade ≥3	0	–	1 (4.5)	1 (5.6)	1 (4.3)	–	–	3 (3.4)
Dyspnea	1 (25.0)	2 (50.0)	2 (9.1)	3 (16.7)	3 (13.0)	2 (16.7)	2 (50.0)	15 (17.2)
Grade ≥3	0	0	0	0	1 (4.3)	0	0	1 (1.1)
Nausea	0	1 (25.0)	3 (13.6)	4 (22.2)	6 (26.1)	0	1 (25.0)	15 (17.2)
Grade ≥3	–	0	1 (4.5)	1 (5.6)	1 (4.3)	–	0	3 (3.4)
Abdominal pain	0	1 (25.0)	2 (9.1)	3 (16.7)	5 (21.7)	2 (16.7)	1 (25.0)	14 (16.1)
Grade ≥3	–	0	1 (4.5)	0	2 (8.7)	2 (16.7)	0	5 (5.7)
Back pain	1 (25.0)	0	4 (18.2)	2 (11.1)	5 (21.7)	1 (8.3)	0	13 (14.9)
Grade ≥3	0	–	1 (4.5)	0	1 (4.3)	0	–	2 (2.3)
Cough	0	1 (25.0)	4 (18.2)	5 (27.8)	1 (4.3)	1 (8.3)	1 (25.0)	13 (14.9)
Grade ≥3	–	0	0	0	0	0	0	0
Diarrhea	1 (25.0)	0	3 (13.6)	4 (22.2)	4 (17.4)	1 (8.3)	0	13 (14.9)
Grade ≥3	0	–	0	0	2 (8.7)	0	–	2 (2.3)
Anemia	0	0	4 (18.2)	2 (11.1)	1 (4.3)	2 (16.7)	2 (50.0)	11 (12.6)
Grade ≥3		–	1 (4.5)		0	1 (8.3)	1 (25.0)	3 (3.4)
Arthralgia	0	3 (75.0)	3 (13.6)	1 (5.6)	2 (8.7)	1 (8.3)	0	10 (11.5)
Grade ≥3	–	0	0	0	1 (4.3)	0	–	1 (1.1)
Fever	0	2 (50.0)	1 (4.5)	0	3 (13.0)	1 (8.3)	1 (25.0)	8 (9.2)
Grade ≥3	–	0	0	–	1 (4.3)	0	0	1 (1.1)
Edema peripheral	0	0	1 (4.5)	3 (16.7)	0	2 (16.7)	1 (25.0)	7 (8.0)
Grade ≥3	–	–	0	0	–	0	0	0
Weight decreased	2 (50.0)	1 (25.0)	0	2 (11.1)	2 (8.7)	0	0	7 (8.0)
Grade ≥3	0	0	–	0	0	–	–	0
Dyspnea exertional	0	0	1 (4.5)	1 (5.6)	0	2 (16.7)	2 (50.0)	6 (6.9)
Grade ≥3	–	–	0	0	–	0	0	0
Pruritus	0	0	2 (9.1)	2 (11.1)	2 (8.7)	0	0	6 (6.9)
Grade ≥3	–	–	0	0	0	–	–	0
Rash	0	0	2 (9.1)	2 (11.1)	1 (4.3)	0	1 (25.0)	6 (6.9)
Grade ≥3	–	–	0	0	0	–	0	0
Blood creatinine increased	1 (25.0)	0	0	1 (5.6)	2 (8.7)	1 (8.3)	0	5 (5.7)
Grade ≥3	0	–	–	0	1 (4.3)	0	–	1 (1.1)
Myalgia	1 (25.0)	2 (50.0)	0	1 (5.6)	0	1 (8.3)	0	5 (5.7)
Grade ≥3	1 (25.0)	0	–	0	–	0	–	1 (1.1)

Patients were counted once under each MedDRA preferred term.

MedDRA, Medical Dictionary for Regulatory Activities.

Serious TEAEs were reported in 34 patients (39.1%). Forty-one patients (47.1%) experienced ≥1 TEAE grade 3 or higher during the study; most common was abdominal pain reported by 5 patients (5.7%); and small intestinal obstruction was reported by 4 patients (4.6%). Seventeen immune-related AEs were reported in 13 patients (14.9%) and included fatigue (n=4), rash (n=3), pruritus (n=3), and 1 each of diarrhea, arthralgia, colitis, eczema, nausea, chills, and musculoskeletal pain. All immune-related events, except for colitis, were grade 1/2. The patient with colitis (received INCAGN01949 at 350 mg) experienced a grade ≥3 immune-related AE on day 7, which met the definition for a DLT; this patient had received avelumab (anti-PD-L1) approximately 2 months prior to receiving the first dose of INCAGN01949.

Eight patients (9.2%) died during the study, all due to tumor progression unrelated to study treatment. Owing to the lack of pharmacodynamic activity of INCAGN01949 and early termination of the study, no MTD or PAD was able to be defined.

### Pharmacokinetics

The mean pharmacokinetic profiles of INCAGN01949 at cycle 1 (post first dose) and cycle 6 (steady state, stratified by dose), and corresponding pharmacokinetic parameters after first dose and steady state are summarized in table 3 (see online supplemental figure S1 for pharmacokinetic profile plots). The mean terminal half-life of INCAGN01949 after the first dose varied between 186 hours (200 mg dose group, n=17) and 313 hours (20 mg dose group, n=4), and between 70 hours (700 mg dose group, n=1) and 289 hours (200 mg dose group, n=3) at steady state.

**Table 3:** Pharmacokinetics of INCAGN01949

Dose level	T_max_ (hour) Median (min, max)	C_max_ (ng/mL) Mean (SD)	AUC_0–t_ (μg·hour/mL)* Mean (SD)	AUC_0–∞_ (μg·hour/mL)* Mean (SD)	Half-life (hour) Mean (SD)	CL/F (L/hour) Mean (SD)	V_z_/F (liter) Mean (SD)
After the first dose (n=78)							
7 mg (n=4)	0.64 (0.6, 4.4)	1630 (735)	185 (81.7)	301 (108)	206 (95.8)	2.71×10^−5^ (1.44×10^−5^)	0.0071 (0.0024)
20 mg (n=4)	2.53 (0.55, 4.5)	5820 (324)	904 (197)	2290 (2460)	313 (313)	1.50×10^−5^ (7.87×10^−6^)	0.0041 (0.0042)
70 mg (n=21)	4.3 (0.50, 25.2)	22,300 (32,800)	2370 (841)	3180 (1060)	198 (71.0)	2.41×10^−5^ (7.19×10^−6^)	0.0066 (0.0023)
200 mg (n=17)	0.63 (0.5, 4.5)	39,200 (10,300)	5400 (1820)	8190 (4030)	186 (90.5)	3.14×10^−5^ (2.00×10^−5^)	0.0075 (0.0039)
350 mg (n=21)	0.6 (0.0, 194)	84,900 (76,700)	10,000 (5600)	18,300 (10,100)	269 (191)	2.40×10^−5^ (1.11×10^−5^)	0.0077 (0.0034)
700 mg* (n=7)	0.7 (0.53, 4.2)	207,000 (45,500)	28,300 (7720)	40,400 (12,000)	197 (45.6)	1.85×10^−5^ (4.80×10^−6^)	0.0050 (0.00088)
1400 mg (n=4)	0.73 (0.0, 2.43)	347,000 (130,000)	61,600 (9130)	102,000 (24,600)	234 (65.4)	1.43×10^−5^ (3.87×10^−6^)	0.0046 (0.0046)
Steady state (n=15)^†^							
7 mg (n=2)	0.78 (0.75, 0.8)	2950 (163)	427 (9.21)	NR	176 (53.2)	1.64×10^−5^ (3.54×10^−7^)	0.0042 (0.0011)
70 mg^‡^ (n=4)	0.63 (0.50, 4.3)	27,800 (8070)	5490 (1560)	NR	251 (80.9)	2.51×10^−5^ (1.62×10^−5^)	0.011 (0.0062)
200 mg^‡^ (n=3)	0.61 (0.55, 4.8)	59,200 (24,500)	10,700 (6560)	NR	289 (101)	1.31×10^−5^ (3.44×10^−6^)	0.00424 (NR)

*One patient was excluded from the pharmacokinetic summary because he/she only had pharmacokinetic sampling from 0 to 4 hours post dose.

†Patients received INCAGN01949 as an intravenous infusion on day 1 of each 14-day (±3) treatment cycle; steady state occurred around cycle 6. The dose levels of 20, 350, and 700 mg are not shown because only one patient had data available at steady state. The 1400 mg dose level is not shown because no patients were enrolled.

‡One patient was excluded since only one pharmacokinetic timepoint sample was collected.

AUC_0−∞_, area under the plasma concentration−time curve extrapolated to time infinity; AUC_0−t_, area under the concentration−time curve up to the last measurable concentration; CL/F, apparent oral dose clearance; C_max_, maximum plasma drug concentration; NR, not reported; SD, standard deviation; T_max_, time to maximum plasma drug concentration; V_z_/F, apparent oral dose volume of distribution.

The main secondary pharmacokinetic endpoints were C_max_, T_max_, and AUC_0-t_. At the dose levels of 7, 70, and 200 mg, median T_max_ values were 0.78, 0.63, and 0.61 hours, respectively; means for C_max_ and AUC_0-t_ were 2950, 27,800, and 59,200 ng/mL, and 427, 5490, and 10,700 µg·hour/mL, respectively. INCAGN01949 exhibited a dose-proportional C_max_ or area under the concentration-time curve across all dose levels tested. Although there were limited data at later timepoints, trough concentrations appeared to increase until cycles 6 and 7, suggesting that steady state occurred around cycle 6 (common observation with antibody-based therapies). Notably, there were no treatment-emergent ADAs detected across all dose levels.

### Efficacy

**Figure 2:**
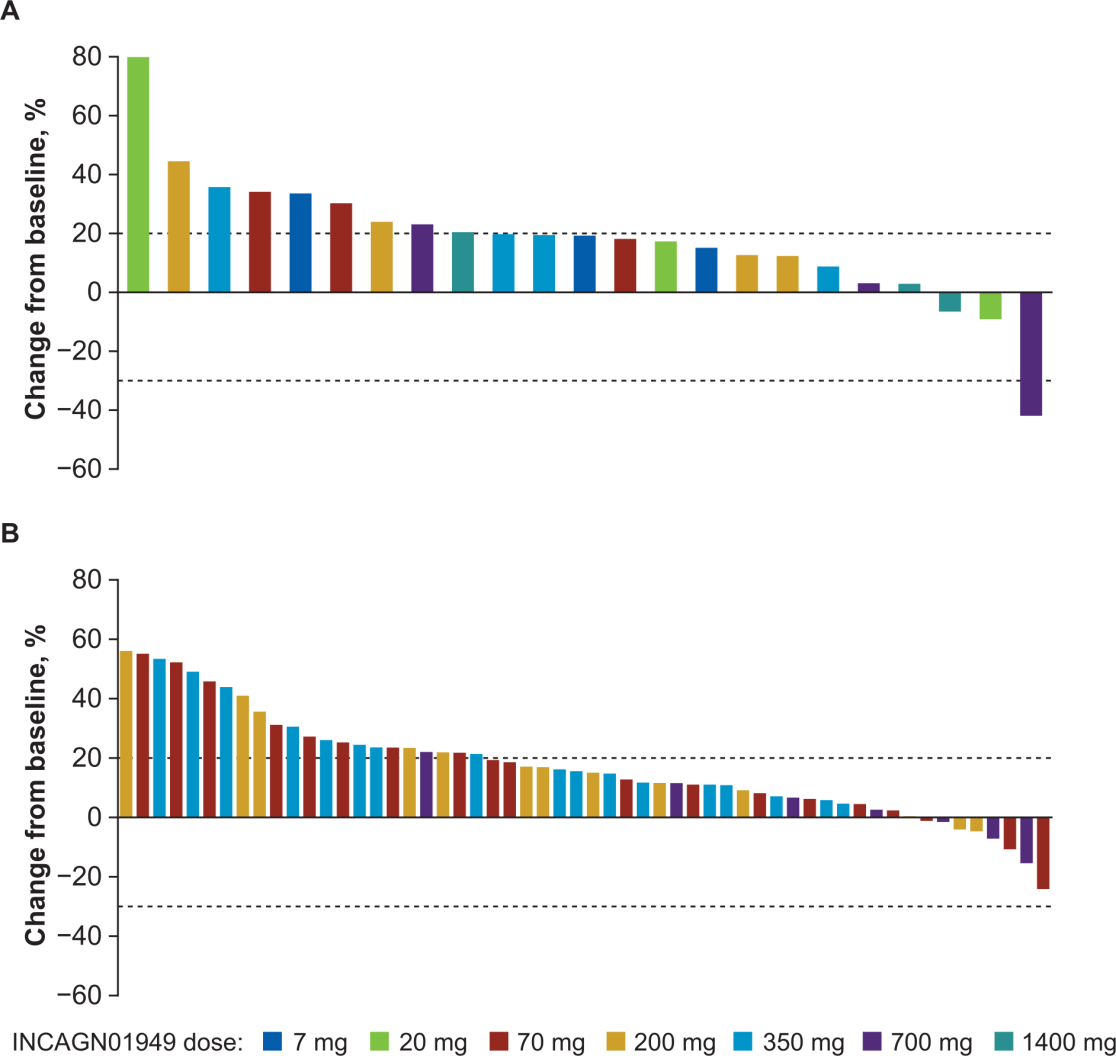
Best percentage change from baseline in target lesion size for individual patients in (A) dose-escalation and (B) safety-expansion populations. Upper limit of dotted line indicates criteria for progressive disease (≥20% increase in sum of target lesion diameters), and lower limit indicates criteria for partial response (≥30% decrease in sum of target lesion diameters).

No CRs were observed in this patient population treated with INCAGN01949. Overall disease control rate (CR, PR, or SD) was 27.6% (24 of 87 patients enrolled). One patient (1.1%) with metastatic gallbladder cancer receiving 700 mg INCAGN01949 demonstrated a PR as best response. The patient’s duration of response was 192 days, from day 106 (after the eighth administration) to day 298 (last response assessment), with largest decrease in tumor size from baseline of 41.9% recorded on day 298. The best percentage change in tumor lesion size for individual patients is shown in figure 2. Twenty-three patients (26.4%) achieved SD (mean duration 81.1 (range 1–225) days) as best response while receiving treatment with INCAGN01949. One patient had SD lasting longer than 6 months. Cancer types in the 23 patients who achieved SD included head and neck cancer, non-small cell lung cancer, and ovarian cancer (n=3 each); colorectal and testicular (n=2 each) cancers; adrenal, breast, chordoma, esophageal, gallbladder, hepatocellular, melanoma, and salivary gland (n=1 each) cancers; and other (n=2).

### Pharmacodynamics

INCAGN01949 receptor occupancy was assessed in an in vitro OX40-expressing cell model, which demonstrated that occupancy was maintained above 90% among all patients receiving doses of ≥200 mg INCAGN01949. Receptor occupancy did not reach full saturation at doses ≤70 mg, with receptor saturation occupancy of <90% at trough (data not shown). Blood levels of soluble OX40 receptor levels increased following treatment (online supplemental figure S2), indicating target engagement by INCAGN01949, which in turn increased receptor half-life.

**Figure 3:**
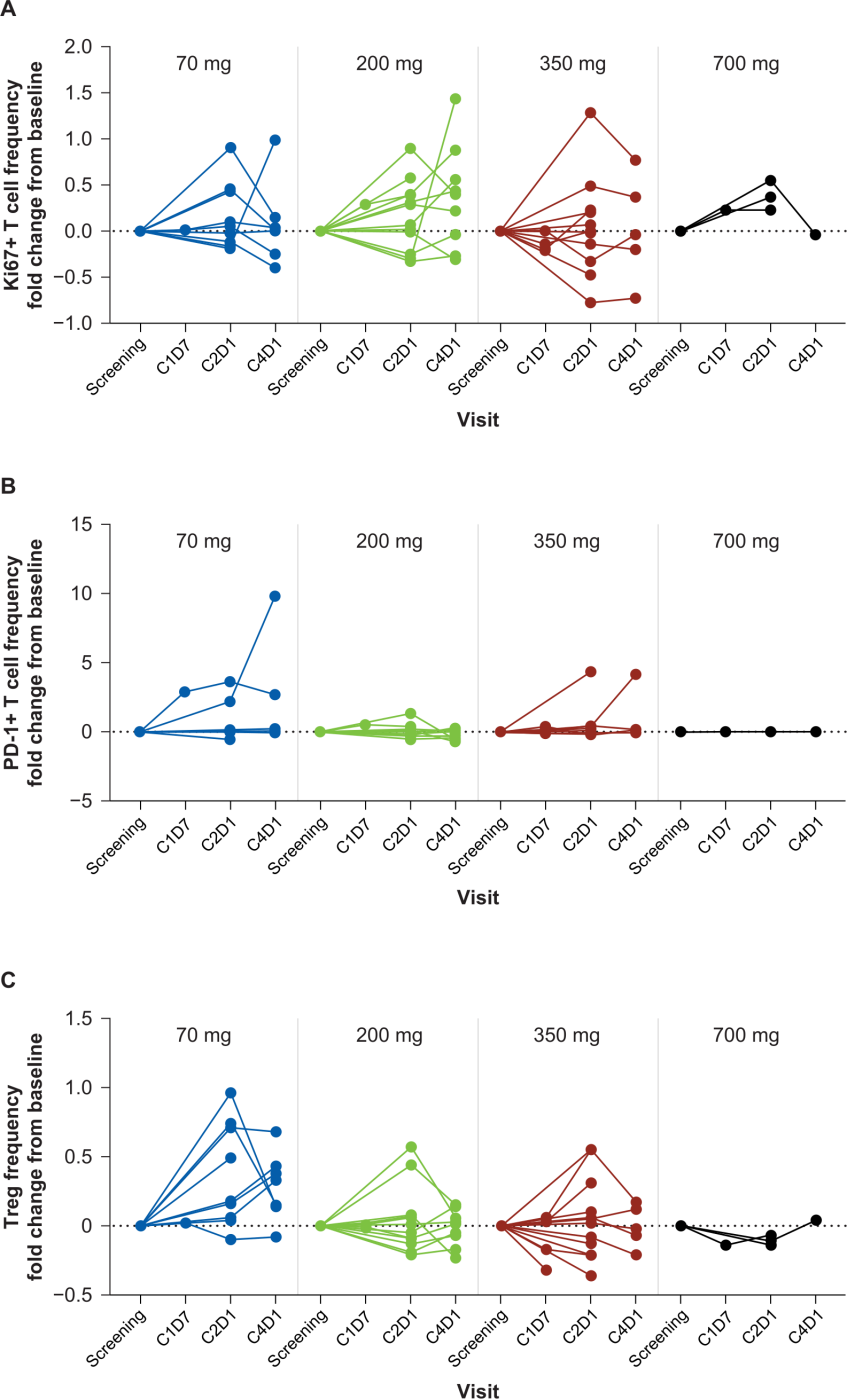
T-cell activity in blood samples of patients treated with INCAGN01949. Fold change from baseline of (A) proliferating (Ki67^+^) T cells. (B) Activated/exhausted (PD-1^+^) T cells. (C) Tregs. C, cycle; D, day; PD-1, programmed cell death protein 1; Treg, regulatory T cell.

**Figure 4:**
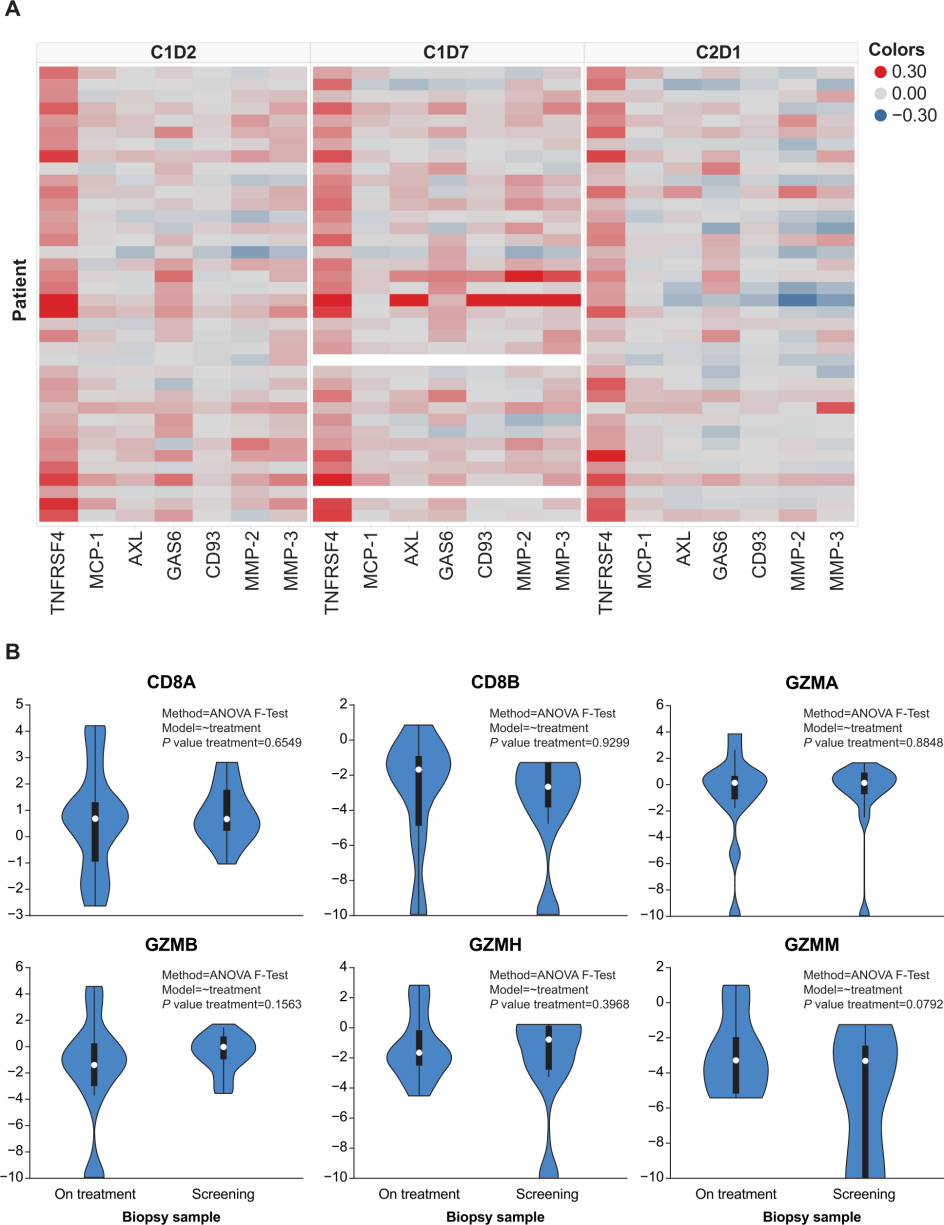
Plasma protein analysis and transcriptomic analysis in tumor samples of patients treated with INCAGN01949. (A) Heat map of immune-related plasma protein expression. Log2-fold change in plasma proteins identified to be differentially expressed between C1D1 and either C1D2, C1D7, and/or C2D1 in the cohorts of 70, 200, and 350 mg. (B) Violin plot of biopsy CD8^+^ T cell-related gene expression. Gene expression was analyzed in biopsy samples collected at screening (pretreatment) and on treatment. Statistical significance was calculated using ANOVA F-test; genes depicted reached statistical significance (p<0.05). ANOVA, analysis of variance; C, cycle; D, day; GZMA, granzyme A; GZMB, granzyme B; GZMH, granzyme H; GZMM, granzyme M.

Treatment with INCAGN01949 did not enhance the proliferation or activation of T cells (total or subtypes) in peripheral blood or decrease the number of circulating Tregs (figure 3). The overall functional status of circulating T cells at early timepoints also remained unaffected by treatment as assessed by cytokine production at a single-cell level following in vitro restimulation (online supplemental figure S3). Plasma protein analysis (figure 4A) demonstrated a significant on-treatment increase in soluble OX40 (TNFRSF4), reflecting soluble OX40 receptor-INCAGN01949 antibody complexes. Other plasma proteins identified as differentially expressed between baseline and any time thereafter suggest a minor increase in immune activation not associated with T-cell activation (figure 4A). Transcriptomic-based analyses of T-cell infiltration and immune activation in tumor biopsies did not demonstrate any consistent increase in effector T-cell infiltration or function (figure 4B), or decrease in Tregs, with INCAGN01949. All analyses of pharmacodynamic activity were validated and shown to be suitable in detecting changes.

## Discussion

This was a first-in-human study with INCAGN01949, designed to evaluate the safety and tolerability in patients with advanced solid tumors. INCAGN01949 was generally well tolerated; TRAEs occurring in ≥5% of patients were fatigue (18%), rash, and diarrhea (7% each) with no dose dependency observed. The overall safety profile was consistent with phase I studies of other OX40 agonist antibodies^24–27^ where fatigue and influenza-like symptoms were among the most common TRAEs. One patient in our study who received anti-PD-L1 therapy about 2 months before their first dose of INCAGN01949 (350 mg) experienced a DLT (colitis). It is unclear whether the colitis was a late immune-related AE associated with the prior anti-PD-L1 treatment or due to INCAGN01949. The MTD was not reached and, as discussed further, a PAD was not determined. Higher doses may have a negative impact on tumor efficacy due to the risk of T-cell overstimulation and exhaustion, or receptor binding and oligomerization.^28^ A similar observation has been made with other OX40 agonists,^27 29^ and this was the rationale for not continuing further dose escalation in our study when the MTD was not reached.

As safety and tolerability were the primary endpoints for this study, the inclusion criteria for part 1 was advanced or metastatic solid tumors without regard to immune-responsive tumor types. It is noteworthy that the radiological PR was observed in a patient with metastatic gallbladder cancer, a tumor type not considered immunologically responsive. The overall disease control rate in our study was 27.6% (1 PR and 23 SDs among 87 patients enrolled). Patients with advanced solid tumors achieving SD have also been reported with other OX40 agonist antibodies, but objective responses are rare in the advanced cancer setting.^24 25 27^ BMS-986178 (a fully human OX40 agonist IgG1 mAb) has also been tested in combinations with nivolumab (an anti-PD-1 antibody) and/or ipilimumab (an anti-CTLA-4 antibody) in advanced solid tumors.^26^ Although no responses were observed with monotherapy, one patient (bladder cancer) receiving combination treatment with nivolumab every 2 weeks achieved a CR.^26^ PRs were seen in 0%–13% among cohorts receiving combination treatments with no clear signal for improved efficacy with the addition of the OX40 agonist.^26^

In preclinical studies, INCAGN01949 maintained a sigmoidal dose–response curve across a range of antibody concentrations, suggesting a broad therapeutic window that may translate to dosing advantages.^23 30^ In addition, selective depletion of OX40-high intratumoral Tregs by the IgG1 Fc region of INCAGN01949 was thought to confer an important secondary mechanism contributing to antitumor activity in preclinical studies.^23 30^ In murine tumor models, there is an optimal immunological dose that generates antitumor activity, with low and high doses showing less robust tumor control.^28 31^ Our results suggest INCAGN01949 monotherapy did not enhance immune activation in advanced solid tumors at the doses tested, indicated by the lack of increase in effector T-cell infiltration or decrease in frequency of Tregs in the tumor microenvironment. Furthermore, no immune activation correlates (increased T-cell activity) in the blood were observed. Levels of LAG3^+^ and TIM3^+^ T cells were below the limit of quantification by flow cytometry, and gene expression analyses were not performed due to limited available specimens. Samples from the one responder were not available for correlative analysis. Although the available number of paired biopsies should be considered a study limitation, the lack of pharmacodynamic activity observed led to the conclusion that patients would not derive clinical benefit, even at higher doses. The study was therefore discontinued and part 2 was not opened.

Reports of pharmacodynamic activity of OX40 agonists in early studies have been variable, with several agents showing some degree of proliferation/activation of peripheral central and memory T-cell subsets.^24 25 27^ Although early results suggested pharmacodynamic effects of BMS-986178 monotherapy, there were no consistent changes in TIL, proliferating CD8^+^ T cells, Tregs, or clinical activity at the end of the study,^26^ consistent with our observations with INCAGN01949.

The relative paucity of OX40-expressing T cells in the peripheral blood may account for failure to detect T-cell activation in the circulation. Approaches to upregulate OX40 expression in the tumor microenvironment may enhance treatment effects and demonstrate greater benefit with OX40 agonists^32^; a study of INCAGN01949 in combination with CMP-001 (toll-like receptor nine agonist; NCT04387071) is planned to investigate such an approach. OX40 plays a major role in CD4 T-cell priming and regulating T-cell differentiation.^33^ As a result, the most significant effect of anti-OX40 monoclonal antibodies could be expected to be observed in the lymphatic tissue. However, this phase I study was primarily designed to assess safety, and a comprehensive analysis with serial collection of lymph node tissue pretreatment and post-treatment to evaluate the effect of the drug in secondary lymphoid organs was beyond the scope of the trial.^33^

Immune checkpoint inhibitors are an important treatment option for patients with cancer and remain a major focus of clinical research. A number of trials with OX40 agonists are ongoing in a range of tumor types, evaluating the therapeutic potential of single agents or in combination therapy with other agents.^24 25 34 35^ In the current study, although there were no safety concerns with INCAGN01949 monotherapy in patients with metastatic or advanced solid tumors, the study was discontinued owing to lack of anticipated pharmacodynamic effects on T cells in peripheral blood and post-therapy tumor biopsies, and lack of clinical activity. Optimizing the clinical effect of OX40 agonism may require novel combination regimens, sequential dosing strategies, or evaluation in selected patients with inflamed tumors.^36 37^ Further studies are needed to elucidate the therapeutic potential of OX40 agonism for the treatment of advanced cancers.

## Data Availability

No data are available.

## Data availability statement

Access to individual patient-level data is not available for this study.
