# Supplementary material for: First-in-human phase I/II, open-label study of the anti-OX40 agonist INCAGN01949 in patients with advanced solid tumors
Source: J Immunother Cancer. 2022 Oct 31;10(10):e004235. doi: 10.1136/jitc-2021-004235 (PMC9628691; doi:10.1136/jitc-2021-004235)

SUPPLEMENTAL MATERIALS

First-in-human phase 1/2, open-label study of the anti-OX40 agonist INCAGN01949 in patients with advanced solid tumors

Elizabeth J Davis[1], Juan Martin-Liberal[2], Rebecca Kristeleit[3*], Daniel C Cho[4†], Sarah P Blagden[5], Dominik Berthold[6], Dana B Cardin[1], Maria Vieito[2], Rowan E Miller[7], Prashanth Hari Dass[8‡], Angela Orcurto[6], Kristen Spencer[9], John E Janik[10¶], Jason Clark[11¶], Thomas Condamine[12¶], Jennifer Pulini[13], Xuejun Chen[13], Janice M Mehnert[14#]

[1]Vanderbilt University Medical Center, Nashville, USA (elizabeth.j.davis@vumc.org, dana.cardin@vumc.org)

[2]Vall d'Hebron Institute of Oncology (VHIO), Barcelona, Spain (jmartinliberal@gmail.com, mvieito@vhio.net)

[3]Guy’s and St. Thomas’ NHS Foundation Trust, London, UK (rebecca.kristeleit@gstt.nhs.uk))

[4]New York Medical College, Westchester Medical Center, Valhalla, NY, USA (Daniel.Cho@wmchealth.org)

[5]Department of Oncology, University of Oxford, Oxford, UK (sarah.blagden@oncology.ox.ac.uk)

[6]Department of Oncology, Centre Hospitalier Universitaire Vaudois (CHUV), Lausanne, Switzerland (Dominik.Berthold@chuv.ch, Angela.Orcurto@chuv.ch)

[7]University College London Hospital, London, UK (rowan.miller1@nhs.net)

[8]Lakes District Health Board, Rotorua, New Zealand (Prashanth.HariDass@lakesdhb.govt.nz)

[9]Rutgers University, New Brunswick, USA (spencekr@cinj.rutgers.edu)

[10]Cullinan Oncology, Cambridge, USA

[11]AstraZeneca, West Chester, USA

[12]Carisma Therapeutics, Philadelphia, USA (Thomas.Condamine@carismatx.com)

[13]Incyte Corporation, Wilmington, USA (jpulini@incyte.com, xchen@incyte.com)

[14]Perlmutter Cancer Center, NYU Langone Health, NYU Grossman School of Medicine, New York, USA (janice.mehnert@nyulangone.org)

Affiliation during the conduct of the study: *Research Department of Oncology, University College London, London, UK; [†]Perlmutter Cancer Center, NYU Langone Health, NYU Grossman School of Medicine, New York, USA; [‡]Early Phase Clinical Trials Unit, Churchill Hospital, University of Oxford, Oxford, UK; [¶]Incyte Corporation, Wilmington, USA; [#]Rutgers University, New Brunswick, USA

Correspondence: Janice M. Mehnert, Perlmutter Cancer Center of NYU Langone Health, NYU Grossman School of Medicine, 160 East 34th Street, 9th Floor, New York, NY 10016 Tel: 212-731-5431; Email: Janice.Mehnert@nyulangone.org

Supplemental Table S1 Definition of DLT (28-day DLT observation period)

**Nonhematologic toxicity**<br>• Any grade ≥3 nonhematologic toxicity EXCEPT the following:<br>– Transient (≤72 hours) abnormal laboratory values without associated clinically significant signs or symptoms<br>– Nausea, vomiting, and diarrhea adequately controlled with supportive care within 48 hours<br>– Changes in cholesterol and triglycerides<br>– An event clearly associated with the underlying disease, disease progression, a concomitant medication, or comorbidity<br>– Asymptomatic changes in lipid profiles<br>– Asymptomatic changes in amylase and lipase<br>– Singular or nonfasting elevations in blood glucose (ie, blood glucose excursions will be considered toxicities if fasting blood glucose is elevated on two separate occasions)
**Hematologic toxicity**<br>• Grade 3 thrombocytopenia with clinically significant bleeding (ie, requires hospitalization, transfusion of blood products, or other urgent medical intervention)<br>• Grade 4 thrombocytopenia<br>• Grade ≥3 febrile neutropenia (absolute neutrophil count <1.0×10^9/L and fever >101°F/38.3°C)<br>• Grade 4 neutropenia that does not recover to grade ≤2 in ≤3 days after interrupting study drug<br>• Grade 4 anemia not explained by underlying disease or some other concomitant disorder
**Immune-related toxicity**[a]<br>• Grade ≥2 ocular irAEs will be considered a DLT<br>• Grade 3 irAEs that do not improve to baseline or at least grade 1 in <5 days with appropriate care or with corticosteroid therapy will be considered a DLT<br>• Grade 4 irAEs will be considered a DLT regardless of duration

**General**
• Inability to receive the planned number of doses within the 28-day DLT period due to toxicity, regardless of grade, will be considered a DLT
**MTD**
• In Part 1 of the study, the MTD will be defined as one dose level below that at which ≥ one-third of patients in a particular cohort have DLTs<br>• In Part 2 of the study, toxicities will continue to be monitored. If the cumulative incidence of DLTs occurs in ≥33% of patients after 6 patients have been observed for at least 28 days, further enrollment may be interrupted, and the investigators and sponsor will meet and reassess the MTD. All AEs, regardless of the time of occurrence on study, may be considered in DLT determination purposes
**MNTD**
• For each dose and schedule explored, if >33% of patients (minimum of 6 patients) experience a grade ≥3 toxicity related to study drug after completing ≥4 cycles, then treatment will be stopped and the MNTDs will be determined in conjunction with the investigators and sponsor based on all available safety data. All AEs, regardless of the time of occurrence on study, may be considered in DLT determination decisions

Transient (≤72 hours) abnormal laboratory values without associated clinically significant signs or symptoms based on investigator determination will not be considered DLTs.

[a]irAEs are a diagnosis of exclusion, after alternative etiologies have been ruled out.

AE, adverse event; DLT, dose-limiting toxicity; irAE, immune-related adverse event; MTD, maximum tolerated dose; MNTD, maximum number of tolerated doses.

Davis EJ, *et al. J Immunother Cancer* 2022; 10:e004235. doi: 10.1136/jitc-2021-004235

Supplemental Figure S1 Mean (± standard error) INCAGN01949 concentration over time after the first dose (cycle 1) and at steady state (cycle 6).

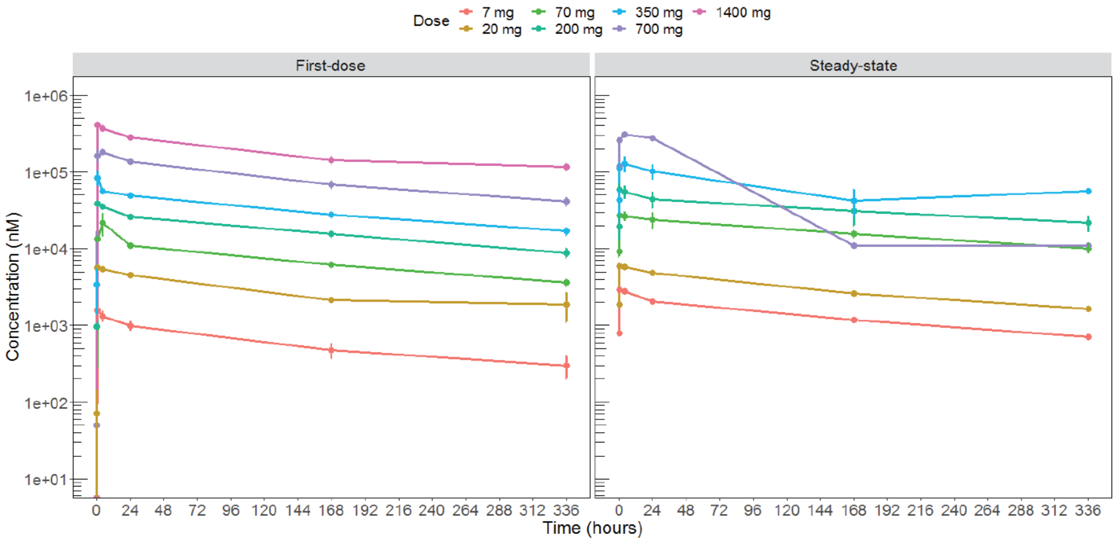

Davis EJ, *et al. J Immunother Cancer* 2022; 10:e004235. doi: 10.1136/jitc-2021-004235

Supplemental Figure S2 Soluble OX40 receptor (TNFRSF4) levels in the plasma of patients treated with INCAGN01949. C, cycle; D, day.

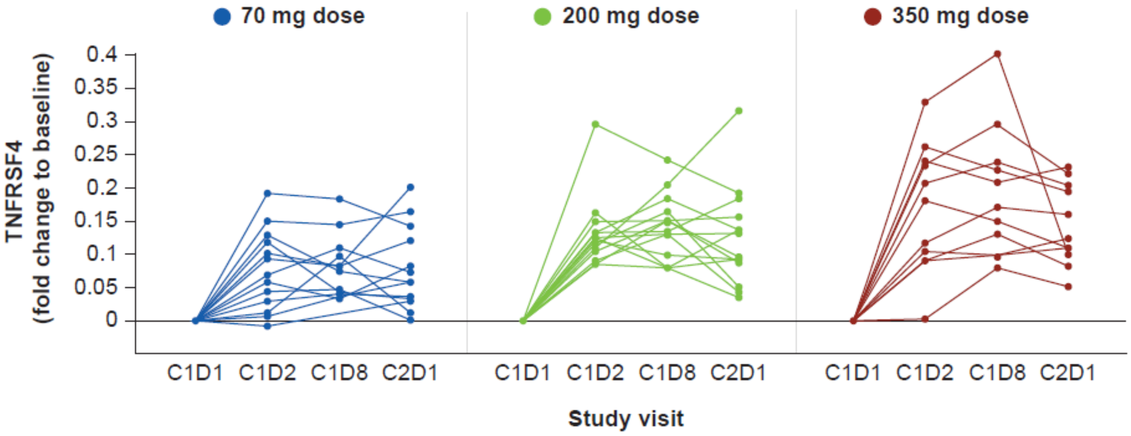

Davis EJ, *et al. J Immunother Cancer* 2022; 10:e004235. doi: 10.1136/jitc-2021-004235

Supplemental Figure S3 Polyfunctional frequency (PFF) and polyfunctional strength index (PSI) of CD4⁺ and CD8⁺ T cells before and after INCAGN01949 treatment. C, cycle; D, day.

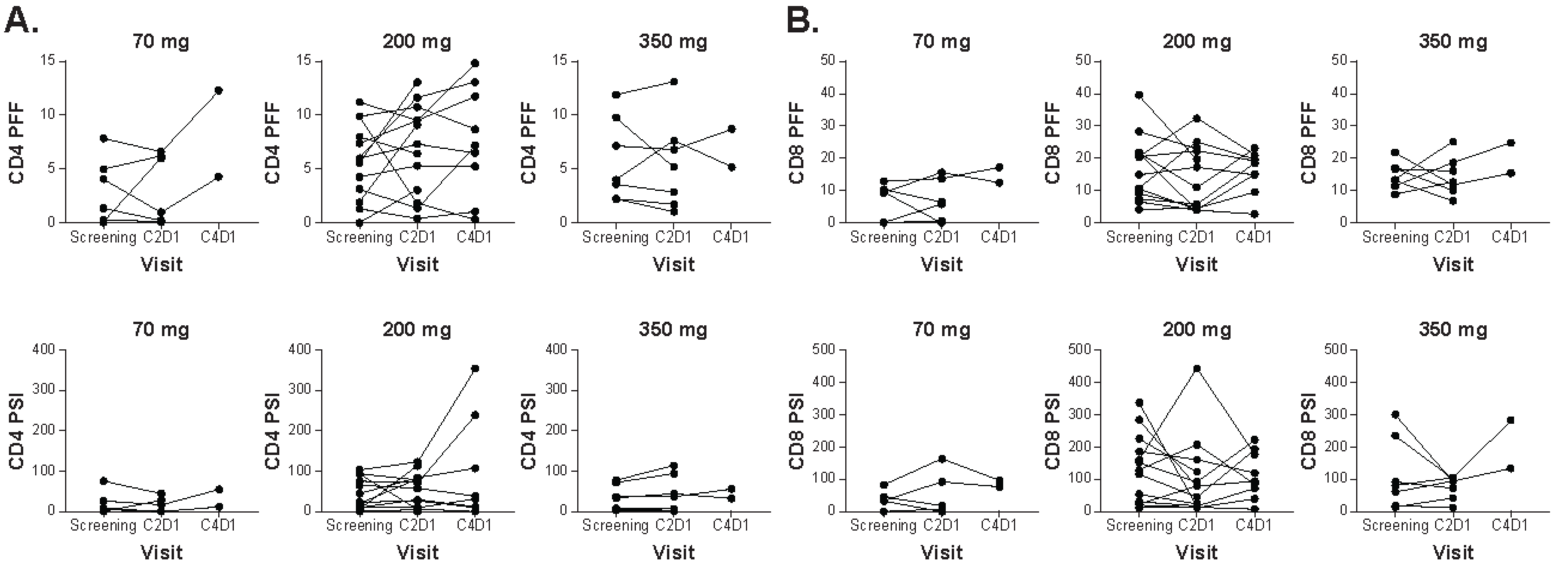

Supplement: Supplementary data [file jitc-2021-004235supp001.pdf]
